# Inhibition of Gα_s_/cAMP Signaling Decreases TCR-Stimulated IL-2 transcription in CD4^+^ T Helper Cells

**DOI:** 10.5334/1750-2187-10-2

**Published:** 2015-07-06

**Authors:** Thomas R. Hynes, Evan A. Yost, Stacy M. Yost, Cassandra M. Hartle, Braden J. Ott, Catherine H. Berlot

**Affiliations:** Weis Center for Research, Geisinger Clinic, Danville, Pennsylvania, 17822-2623, United States of America

**Keywords:** cAMP, T helper cells, heterotrimeric G-protein, Gα_s_, G-protein-coupled receptor, IL-2

## Abstract

**Background:** The role of cAMP in regulating T cell activation and function has been controversial. cAMP is generally known as an immunosuppressant, but it is also required for generating optimal immune responses. As the effect of cAMP is likely to depend on its cellular context, the current study investigated whether the mechanism of activation of Gα_s_ and adenylyl cyclase influences their effect on T cell receptor (TCR)-stimulated interleukin-2 (IL-2) mRNA levels.

**Methods:** The effect of blocking G_s_-coupled receptor (G_s_PCR)-mediated G_s_ activation on TCR-stimulated IL-2 mRNA levels in CD4^+^ T cells was compared with that of knocking down Gα_s_ expression or inhibiting adenylyl cyclase activity. The effect of knocking down Gα_s_ expression on TCR-stimulated cAMP accumulation was compared with that of blocking G_s_PCR signaling.

**Results:** ZM-241385, an antagonist to the G_s_-coupled A_2A_ adenosine receptor (A_2A_R), enhanced TCR-stimulated IL-2 mRNA levels in primary human CD4^+^ T helper cells and in Jurkat T cells. A dominant negative Gα_s_ construct, Gα_s_DN3, also enhanced TCR-stimulated IL-2 mRNA levels. Similar to GsPCR antagonists, Gα_s_DN3 blocked G_s_PCR-dependent activation of both Gα_s_ and Gβγ. In contrast, Gα_s_ siRNA and 2’,5’-dideoxyadenosine (ddA), an adenylyl cyclase inhibitor, decreased TCR-stimulated IL-2 mRNA levels. Gα_s_ siRNA, but not Gα_s_DN3, decreased TCR-stimulated cAMP synthesis. Potentiation of IL-2 mRNA levels by ZM-241385 required at least two days of TCR stimulation, and addition of ddA after three days of TCR stimulation enhanced IL-2 mRNA levels.

**Conclusions:** G_s_PCRs play an inhibitory role in the regulation of TCR-stimulated IL-2 mRNA levels whereas Gα_s_ and cAMP can play a stimulatory one. Additionally, TCR-dependent activation of Gα_s_ does not appear to involve G_s_PCRs. These results suggest that the context of Gα_s_/cAMP activation and the stage of T cell activation and differentiation determine the effect on TCR-stimulated IL-2 mRNA levels.

## Introduction

The cellular effects of the second messenger cAMP are often dependent on the context of concentration changes. For instance, cAMP stimulates proliferation in certain cell types whereas it inhibits proliferation in others [1,2], which can be determined by the expression of other signaling components [3,4]. A-kinase anchoring proteins (AKAPs) [5] and cAMP-degrading phosphodiesterases (PDEs) [6] can determine the physiological effects of cAMP by regulating the spatial and temporal organization of cAMP pathway components. Differential effects of cAMP can result from the selective involvement of protein kinase A (PKA) or exchange protein directly activated by cAMP (Epac) [7,8], which in turn can be determined by the intensity and localization of upstream signals [9]. Given this complexity of cAMP regulation and effects, it is not surprising that the role of cAMP in regulating T cell activation and function has been controversial. cAMP is generally known as an immunosuppressant, but it is also required for generating optimal immune responses.

On one hand, studies utilizing agonists and antagonists of G_s_PCRs, cAMP analogs, and cholera toxin have demonstrated an inhibitory role of cAMP on T cells. For instance, numerous studies of G_s_PCRs such as the A_2A_R[10,11,12,13], PGE_2_ receptors [14,15], and vasoactive intestinal peptide (VIP) receptors [16,17] have demonstrated inhibition of TCR-stimulated production of IL-2, a growth factor for effector and regulatory T cells that has been used to augment immune responses to treat cancer [18] and persistent viral infections [19], and, at lower doses, to suppress immune responses in chronic graft-versus-host disease [20] and hepatitis C virus-induced vasculitis [21]. Moreover, treatment of cultures of human T lymphocytes and monocytes with forskolin to activate adenylyl cyclase, cAMP phosphodiesterase (PDE) inhibitors, or a cell-permeable cAMP analog inhibited phytohemagglutinin (PHA)-stimulated IL-2 production [22]. Additionally, treatment of proliferating T lymphocytes with cAMP analogs inhibited cell replication [23] and led to phosphorylation and activation of Csk, the most proximal PKA substrate [24]. Overexpression of Csk resulted in decreased levels of IL-2 in Jurkat T cells activated by anti-CD3 antibodies and phorbol 12-myristate 13-acetate (PMA) [24]. Furthermore, treatment of Jurkat cells with cholera toxin, which constitutively activates Gα_s_, inhibited TCR-stimulated increases in inositol trisphosphate (IP_3_) and Ca^2+^ [25].

On the other hand, there is precedent for cAMP playing a positive role in T cell function. Mice that lacked Gα_s_ had reduced cAMP levels, decreased Ca^2+^ influx, and impaired TH1 and TH17 differentiation [26], and T cells from mice that lacked the AC7 isoform of adenylyl cyclase were defective in T cell help and memory function [27]. Moreover, the EP_2_ and EP_4_ receptors for PGE_2_ facilitated TH17 expansion by means of the cAMP/PKA pathway [28], and Gα_s_ activation by cholera toxin induced TH17 cells and protected against inhalation anthrax [29]. Additionally, treatment of mouse spleen cell cultures with low concentrations of dibutyryl cAMP increased humoral immune responses and enhanced PMA/ionomycin-stimulated lymphoproliferation, whereas incubation of the cells with ddA decreased both of these responses in parallel with decreasing basal levels of cAMP [30]. Furthermore, transient adhesion-dependent cAMP increases were stimulatory to TCR signaling, although sustained increases in response to forskolin were inhibitory [31]. The amplitude and duration of cAMP increases may also determine the effect on TCR-stimulated IL-2 synthesis. For instance, antigen stimulation of a murine T cell line produced a transient rise in cAMP that correlated with T cell proliferation and IL-2 production [32]. Moreover, a study in Jurkat T cells suggested that sustained increases in cAMP were required to inhibit PHA-stimulated IL-2 production whereas smaller and transient cAMP increases were not sufficient for inhibition and sometimes even caused increases in IL-2 [33].

Prior studies suggest that the context in which cAMP levels are increased can determine the effect on T cell function. For instance, VIP receptors can inhibit production of IL-2 in T cells stimulated by the TCR or ConA, but not by PMA and the Ca^2+^ ionophore, A23187 [16,17]. Similarly, although forskolin and a cAMP analog inhibited IL-2 production by T cells stimulated by PMA/A23187, the EC_50_ was about 10-fold higher than that in T cells activated by PHA [22]. Additionally, dibutyryl cAMP augmented synergistic stimulation of DNA synthesis in guinea pig lymphocytes by diacylglycerol and low concentrations of A23187 while having an inhibitory effect in the presence of higher concentrations of the ionophore [34].

Given the complexities of the effects of cAMP on immune function in general and IL-2 production in particular, cAMP levels and effects are likely to be controlled by multiple inputs that are integrated according to the cellular context. As a step towards elucidating how the nature of the upstream activation of Gα_s_ and adenylyl cyclase might influence their effect on TCR-stimulated IL-2 mRNA levels, the purpose of the current study was to compare the effects of blocking G_s_PCR-mediated G_s_ activation versus inhibiting cAMP synthesis at the level of Gα_s_ or adenylyl cyclase. We found that the former resulted in increased TCR-stimulated IL-2 mRNA levels in contrast to the latter, which caused decreases. Moreover, cAMP increases stimulated by the TCR were inhibited by Gα_s_ siRNA, but not by a dominant negative Gα_s_ construct, Gα_s_DN3, consistent with the conclusion that the TCR stimulates cAMP synthesis via Gα_s_, but not a G_s_PCR. Taken together, these results suggest that the source and context of activated Gα_s_ and cAMP determine whether they increase or decrease levels of TCR-stimulated IL-2 mRNA.

## Methods

### Plasmids

Gα_s_DN3 was produced as described [35], where it was referred to as α_s_(α3β5/G226A/A366S). Gα_s_DN3-CFP was produced by subcloning an EcoRI fragment from Gα_s_DN3 containing the α3β5, G226A, and A366S mutations in place of the corresponding fragment in Gα_s_-CFP [36]. Gα_s_-YFP was produced as described for Gα_s_-CFP using enhanced YFP containing a substitution of Met for Gln-69 instead of enhanced CFP. All Gα_s_ subunit constructs contain mutations that encode the EE epitope as described [37]. YFP-N-β_1_ and YFP-C-γ_7_ were produced as described [38]. The human HA-tagged β_2_AR cDNA was kindly provided by Brian Kobilka (Stanford University, Stanford, CA). β_2_AR-GFP was produced as described [38]. mRFP-Mem was produced as described [39]. For luciferase reporter assays, a 1 kB sequence encoding the human IL-2 promoter from -950 to +48 bp from Panomics/Affymetrix was subcloned into pGL3 (Promega). pRL-TK Renilla (Promega) was used to normalize luciferase activities. Subcloning and mutagenesis procedures were verified by restriction enzyme analysis and DNA sequencing.

### Ethics statement and study population

This study was reviewed and approved by the Geisinger Health System Internal Review Board, and all study participants signed informed consent. Peripheral blood was obtained from 20 healthy women 18 to 70 years old who did not have any autoimmune, infectious, or atopic diseases, clinical suspicion of anemia, or treatment with greater than 10 mg of prednisone within 12 hour of the blood draw.

### Isolation and culture of human CD4^+^ T cells and Jurkat T cells

Peripheral blood mononuclear cells (PBMCs) were isolated using Ficoll-Paque density gradient centrifugation. CD4^+^ T cells were isolated by depletion of non-CD4^+^ T cells using a CD4^+^ T Cell Isolation Kit II (Miltenyi Biotec). The cells were then separated into naïve and memory CD4^+^ T cells using a Naïve CD4^+^ T cell Isolation Kit (Miltenyi Biotec). Purification of the cells was confirmed by labeling samples before and after purification with fluorescently labeled antibodies to either CD4 and CD45RA (to label naïve cells) or CD4 and CD45RO (to label memory cells) and analysis using flow cytometry. 93.5% of the cells in the naïve T cell preparations were CD4^+^ (SE = 0.8%, ranging from 83.9% to 98.2%) and 84.3% were CD45RA^+^ (SE = 1.6%, ranging from 68.1% to 94.2%). 94.8% of the cells in the memory T cell preparations were CD4^+^ (SE = 0.4%, ranging from 89.7% to 97.4%) and 74.1% were CD45RO^+^ (SE = 2.2%, ranging from 55.0% to 87.3%). Cells were plated at a density of 2–9 × 10^6^ cells/ml (depending on yield) in 24-well dishes coated with 2.5 µg/ml anti-CD3 antibody (Miltenyi) in RPMI containing 10% fetal bovine serum, 2.5 µg/ml anti-CD28 antibody (Miltenyi) and IL-2 (2 ng/ml) (R&D Systems). For TH1 differentiation, the media also included 20 ng/ml IL-12 and 1 µg/ml anti-IL-4 antibody (R&D Systems). For TH2 differentiation, the media also included 20 ng/ml IL-4 and 2 µg/ml anti-IL-12 antibody (R&D Systems). Cells were harvested after 3 days.

Jurkat T cells (Clone E6-1) were obtained from ATCC and cultured in RPMI containing 10% fetal bovine serum. For TCR activation, the cells were grown in wells coated with anti-CD3 (2.5 µg/ml) in the presence of soluble anti-CD28 (2.5 µg/ml).

### ZM-241385, ddA, siRNA, and plasmid treatments

10 µM ZM-241385 and 150 µM ddA were added when the T cells were placed in activating/differentiating media.

siRNAs were produced by Dharmacon. The sequence of Gα_s_ siRNA, CGAUGUGACUGCCAUCAUC, was from [40]. The non-targeting (NT) siRNA used was ON-TARGETplus Non-targeting Pool (Dharmacon, D-001810-10-20). 4 × 10^6^ Jurkat cells were nucleofected with 10 µM siRNA in 100 µl of Cell Line Nucleofector Kit V using Program X-005. After two days, the cells were nucleofected again with siRNA in the same manner and then stimulated or not with plate-bound anti-CD3 and soluble anti-CD28 for 3 days.

4 × 10^6^ Jurkat cells were nucleofected with 3.5 µg of α_s_DN3 or empty vector (pcDNAI/Amp) and then stimulated with plate-bound anti-CD3 and soluble anti-CD28 for 3 days.

### Quantitative PCR (qPCR)

RNA was prepared using RNeasy Plus Mini Kits (Qiagen). cDNA was prepared using QuantiTect Reverse Transcription kits (Qiagen). QPCR was performed using TaqMan Gene Expression Assays (Applied Biosystems) and an Applied Biosystems qPCR machine. mRNA expression levels were determined by comparing the C_t_ value of the mRNA of interest to that of the house-keeping gene GAPDH in the same preparation.

### Imaging of fluorescent fusion proteins

HEK-293 cells (ATCC, CRL-1573) were plated at a density of 10^5^ cells per well on Lab-Tek II, 4 well chambered coverslips and transiently transfected using 0.25 µl of LipofectAMINE 2000 Reagent. Cells were imaged 2 days after transfection at 63 × using a Zeiss Axiovert 200 fluorescence microscope under the control of IPLab software as described [38]. Using the motorized x-y-z stage, time course images of cells located at 5–6 positions in the well were collected simultaneously as described [36]. Images for each color channel and DIC were collected at each position in the well every 60 seconds. Following the second time point, cells were stimulated with 10 µM isoproterenol (final concentration) and images were collected for 30 minutes. For each experimental condition, cells were imaged from plates transfected on 3 different days.

### Image Analysis

Time course images were analyzed using IPLab software. Changes in the plasma membrane intensity of labeled proteins were measured in cells co-expressing a membrane marker (mRFP-Mem) that was used to segment membrane pixels and correct for intensity changes due to changes in cell shape as described [36]. Briefly, a segment of pixels covering a length of the plasma membrane was identified using the image of the membrane marker. The average intensities of these pixels in the background- and bleach-corrected images of the labeled protein and membrane marker were determined. The membrane marker intensity values were normalized to a starting value of one and the labeled protein intensity values were divided by the normalized membrane marker values. The corrected labeled protein intensities were normalized to a starting value of one and averaged with values from multiple cells.

### Immunoblots

Using Jurkat cell membranes prepared as described [41], a polyclonal antibody directed at Gα_s_ residues 28–42 [42], prepared in the laboratory of Henry Bourne (University of California, San Francisco), was used to detect expression of Gα_s_, and Gβ_1_ (XAB-00301-1-G) and Gβ_2_ (XAB-00401-1-G) antibodies from CytoSignal, LLC were used to detect expression of Gβ_1_ and Gβ_2_, respectively. Membrane proteins were resolved on NuPAGE 4–12% Bis-Tris gels and transferred to Invitrolon PVDF membranes (Life Technologies). The antigen-antibody complexes were detected using SuperSignal West Pico Chemiluminescent Substrate (Pierce Biotechnology, Inc.). Chemiluminescence was imaged using a Fuji LAS-4000 Luminescent Image Analyzer. Bands in the images were quantified using ImageJ software. For quantification of Gα_s_, both the long and short forms of Gα_s_ [43] were measured together.

### Actinomycin D assay

Jurkat cells were stimulated with plate-bound anti-CD3 and soluble anti-CD28 for three days in the presence or absence of 150 µM ddA and then treated with 10 µg/mL of Actinomycin D to inhibit transcription. After incubation with Actinomycin D for 0, 10, 20, 30, or 60 minutes, the cells were removed from the wells, RNA was prepared, and IL-2 mRNA levels were determined by qPCR.

### Luciferase Assay

Jurkat cells were nucleofected with 2 µg of a luciferase reporter plasmid and 0.1 µg of pRL-TK Renilla and then stimulated or not with plate-bound anti-CD3 (2.5 µg/mL) and soluble anti-CD28 (2.5 µg/mL) in the presence or absence of 150 µM ddA. The Dual-Luciferase Reporter Assay System (Promega) was used according to the manufacturer’s instructions and data were collected using a POLARstar Optima plate reader.

### cAMP accumulation assay

4 × 10^6^ Jurkat cells were nucleofected with 3.5 µg α_s_DN3 or pcDNAI/Amp and then labeled with 40 µCi of [^3^H]-adenine for 24 hours, or nucleofected twice with siRNAs as described above and then labeled with 40 µCi of [^3^H]-adenine for 24 hours before the assay. On the day of the assay, the cells were pelleted, washed once, and then resuspended in HEPES-buffered RPMI without bicarbonate with 10% fetal bovine serum, and 1 x 10^6^ cells in 0.5 mL were plated per well in triplicate in 24-well plates. For TCR activation, the wells were pre-coated with 2.5 µg/ml anti-CD3 and 2.5 µg/ml soluble anti-CD28 was added to the media. For stimulation of the A_2A_R, 300 µM CGS-21680 was added to the media. The media also contained 1 mM 1-methyl-3-isobutylxanthine, a phosphodiesterase inhibitor. Cells were incubated for 40 minutes at 37^o^C. Reactions were terminated by adding an equal volume of TCA stop buffer (10% TCA, 2 mM ATP, and 2 mM cAMP). Nucleotides were separated on ion exchange columns [44]. cAMP accumulation was determined as 1000 X [^3^H]cAMP/([^3^H]ATP + [^3^H]cAMP). Relative cAMP levels in stimulated cells were expressed as the ratio of the value in stimulated cells to the basal value.

### Statistics

The significance of effects of on primary CD4^+^ T cells was determined using the Wilcoxon signed rank test (paired, non-parametric). The significance of effects on Jurkat T cells was determined using the paired T test. Values of *p* < 0.05 were considered significant (^*^, *p* < 0.05; ^**^, *p* < 0.01; ^***^, *p* < 0.001; ^****^, *p* < 0.0001).

## Results

### Inhibiting the A_2A_R in primary human CD4^+^ T helper cells and Jurkat cells enhances TCR-stimulated IL-2 mRNA increases

As prior reports suggested that the effect of cAMP increases on TCR-stimulated IL-2 synthesis might depend on the nature and context of these increases[16,17,22,32,33], we directly compared the effects of inhibiting different upstream activators of cAMP synthesis in CD4^+^ T helper cells co-stimulated by antibodies to CD3, which associates with the TCR and links it to downstream signaling molecules [45], and CD28, which provides an additional signal that is needed for complete T cell activation and regulation of IL-2 production [46]. The cells were stimulated for three days, an interval during which primary CD4^+^ T cells proliferate and differentiate into polarized phenotypes[47,48,49].

**Figure 1:**
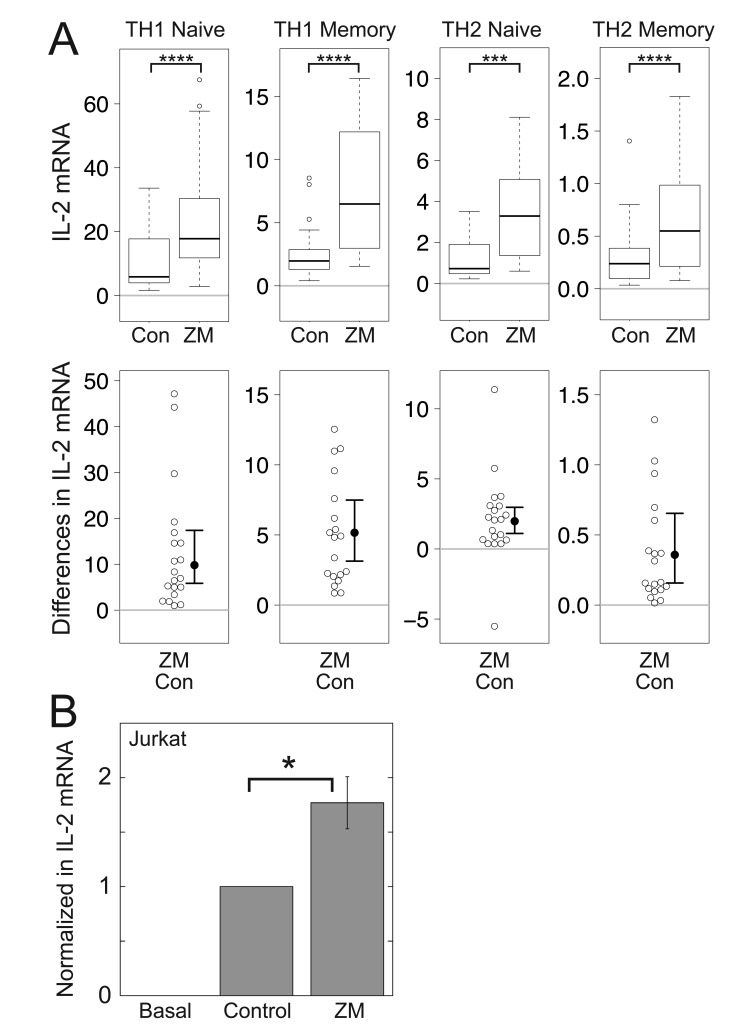
**Antagonism of the A_2A_R enhances TCR-stimulated IL-2 mRNA increases in primary human CD4^+^ T cells and Jurkat T cells.** (A) Box plots (top) and difference plots (bottom) show data from naïve and memory CD4^+^ T cells isolated from the peripheral blood of 20 healthy donors, stimulated with plate-bound anti-CD3 and soluble anti-CD28, and grown in conditions promoting TH1 or TH2 differentiation for three days in the presence or absence of ZM-241385 (ZM). IL-2 mRNA levels were determined by qPCR. In the box plots (top), the height of the box plots equals the interquartile range (IQR) and the horizontal line within the box indicates the median value. The whiskers extend to the lowest and highest data points within 1.5 X IQR and the open circles indicate the outliers, which lie above or below the whiskers. In the difference plots (bottom), open circles show pairwise differences in IL-2 mRNA for each sample when treated with ZM-241385 (ZM) or not (Con). To the right of the open circles are the median values (closed circles) and 95% confidence intervals. (B) Jurkat cells were stimulated with plate-bound anti-CD3 and soluble anti-CD28 in the absence or presence of ZM-241385 (ZM) for three days. IL-2 mRNA levels were determined by qPCR and normalized to the amount produced by the TCR-stimulated control. Data represent the mean ± SE from 8 experiments. ^*^, *p* < 0.05; ^***^, *p* < 0.001; ^****^, *p* < 0.0001.

First, we studied the effect of antagonizing the A_2A_R, which is known to have anti-inflammatory effects mediated by Gα_s_ [50] and can decrease TCR-stimulated IL-2 [10]. ATP released from necrotic and apoptotic cells, regulatory T cells, and effector T cells is converted to adenosine by extracellular ectonucleotidases, or cell surface ectonucleotidases in the case of regulatory T cells, resulting in suppression of T cell function by autocrine or paracrine signaling loops [51]. We tested the effect of ZM-241385 [52], an antagonist to the A_2A_R, on TCR-stimulated IL-2 mRNA increases in primary human CD4^+^ T cells grown in conditions that promote either TH1 or TH2 differentiation and in the Jurkat human CD4^+^ T cell leukemia line, a well-established model system for studying T cell receptor signaling [53] (Fig. 1). We measured IL-2 mRNA by qPCR, as levels of IL-2 are primarily regulated at the level of transcriptional induction of the IL-2 gene and stability of IL-2 mRNA [54,55], and because our own comparisons of qPCR-determined IL-2 mRNA levels and secreted IL-2 [56] and those of others [57] demonstrated a strong correlation between mRNA and protein levels. There was more IL-2 mRNA in TH1 cells than in TH2 cells and in naïve compared to memory cells, as previously reported [56], but ZM-241385 significantly enhanced mean TCR-stimulated IL-2 mRNA levels in each of the primary cell lineages tested, by 1.9 to 3.5-fold, depending on the T cell subset (Fig. 1A), and by 1.8-fold in Jurkat cells (Fig. 1B).

### A dominant negative Gα_s_ construct, Gα_s_DN3, which blocks signaling from G_s_-coupled receptors, enhances TCR-stimulated IL-2 mRNA increases

**Figure 2:**
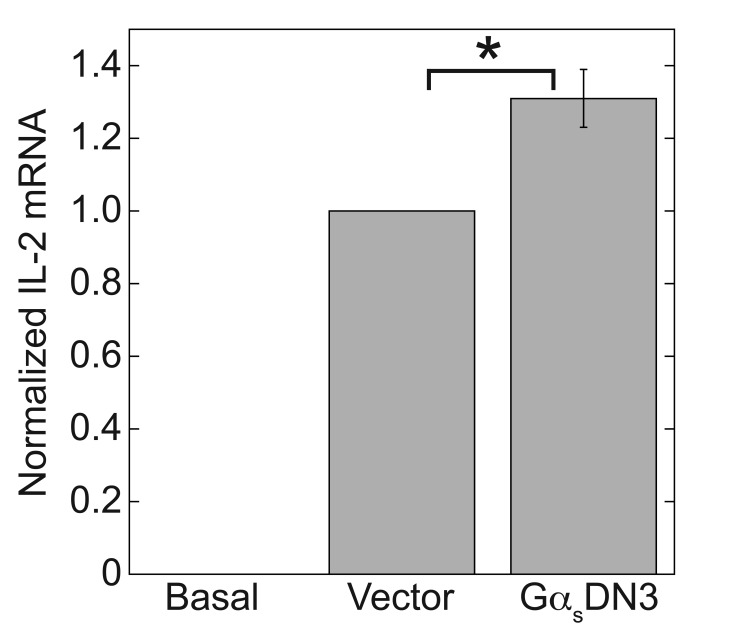
**A dominant negative Gα_s_ construct, Gα_s_DN3, which blocks signaling from G_s_-coupled receptors, enhances TCR-stimulated IL-2 mRNA increases.** Jurkat cells were nucleofected with Gα_s_DN3 or empty vector (pcDNAI/Amp) and then stimulated with plate-bound anti-CD3 and soluble anti-CD28 for 3 days. IL-2 mRNA levels were determined by qPCR and normalized to the amount produced by the TCR-stimulated control. Data represent the mean ± SE from 8 experiments. ^*^, *p* < 0.05.

To determine whether the results of antagonizing the A_2A_R with ZM-241385 could be generalized to other G_s_-coupled receptors under our TCR-activating conditions, we tested the effect of a dominant negative Gα_s_ construct, Gα_s_(α3β5/G226A/A366S), referred to here as Gα_s_DN3, which exhibits increased receptor affinity and blocks stimulation of cAMP synthesis by G_s_PCRs [35,58]. Gα_s_DN3 potentiated the TCR-stimulated increase in IL-2 mRNA by 1.31-fold (Fig. 2). The increased effectiveness of ZM-241385 compared to Gα_s_DN3 is most likely due to the less than 100% efficiency of plasmid expression in nucleofected Jurkat cells.

### Gα_s_DN3 blocks signaling of both Gα_s_ and Gβγ

We observed previously that Gα_s_DN3 inhibited stimulation of cAMP production by G_s_PCRs [35], a Gα_s_-mediated function, but we didn’t determine whether Gβγ function was also inhibited. This was relevant because we determined previously that inhibition of Gβγ by either Gβ_1_ siRNA, which inhibits both Gβ_1_γ and Gα signaling downstream from G protein-coupled receptors (GPCRs), and by gallein, which specifically blocks Gβγ-effector interactions downstream of GPCR-G protein interactions [59], potentiated rather than inhibited TCR-stimulated increases in IL-2 transcription in CD4^+^ T helper cells [56]. Thus, the enhancing effects of both ZM-241385 and Gα_s_DN3 on TCR-stimulated IL-2 mRNA levels might be the result of inhibiting both Gβγ and Gα_s_ together, or Gβγ alone, rather than Gα_s_.

Gα_s_DN3 contains three sets of mutations in Gα_s_, substitutions of Gα_i2_ homologs for Gα_s_ residues in the α3β5 loop, G226A, and A366S [35]. Previously, we demonstrated that the α3β5 loop mutations increased the apparent affinity of Gα_s_ for the β-adrenergic receptor (βAR) [58] using an assay that measures a Gα_s_-dependent increase in the affinity of the βAR for the agonist isoproterenol that occurs in the absence of bound guanine nucleotide [60,61]. The G226A mutation prevents an activating conformational change in Gα_s_ [62,63], and A366S elevates basal GDP release, causing Gα_s_ to be constitutively activated and to spend more time in the empty state [63].

We hypothesized that the combined effects of the α3β5 loop mutations, G226A, and A366S in Gα_s_DN3 might prevent activation of both Gα_s_ and Gβγ derived from G_s_ by causing the formation of a stable receptor-G_s_ complex that does not dissociate upon agonist binding. To investigate this possibility, we imaged the basal and agonist-stimulated localization patterns of the G_s_ subunits and the β_2_AR in the presence and absence of Gα_s_DN3 in live HEK-293 cells expressing fluorescent fusion proteins. We focused on the β_2_AR because we determined previously that activation of the β_2_AR resulted in internalization from the plasma membrane of both Gα_s_ and Gβγ, as well as the β_2_AR itself, allowing us to use internalization as a readout for signaling of each of these components [36]. Previous studies showed that the A_2A_R does not internalize upon prolonged agonist stimulation [64]. Gα_s_ and Gα_s_DN3 were visualized using fusion proteins in which CFP or YFP was inserted into an internal loop of Gα_s_ [36]. Gβ_1_ and Gγ_7_ were imaged exclusively in the form of Gβ_1_γ_7_ complexes using the strategy of bimolecular fluorescence complementation [65,66], which involves the production of a fluorescent signal by two nonfluorescent fragments of YFP or CFP when they are brought together by interactions between proteins fused to each fragment. When expressed together, fusion proteins consisting of an amino-terminal YFP fragment (residues 1–158) fused to Gβ_1_, YFP-N-Gβ_1_, and a carboxy-terminal YFP fragment (residues 159–238) fused to Gγ_7_, YFP-C-Gγ_7_, produce a fluorescent signal in the plasma membrane that is not obtained with either subunit alone [38]. The β_2_AR was visualized using a fusion of GFP to the carboxyl terminus of the β_2_AR [36].

**Figure 3:**
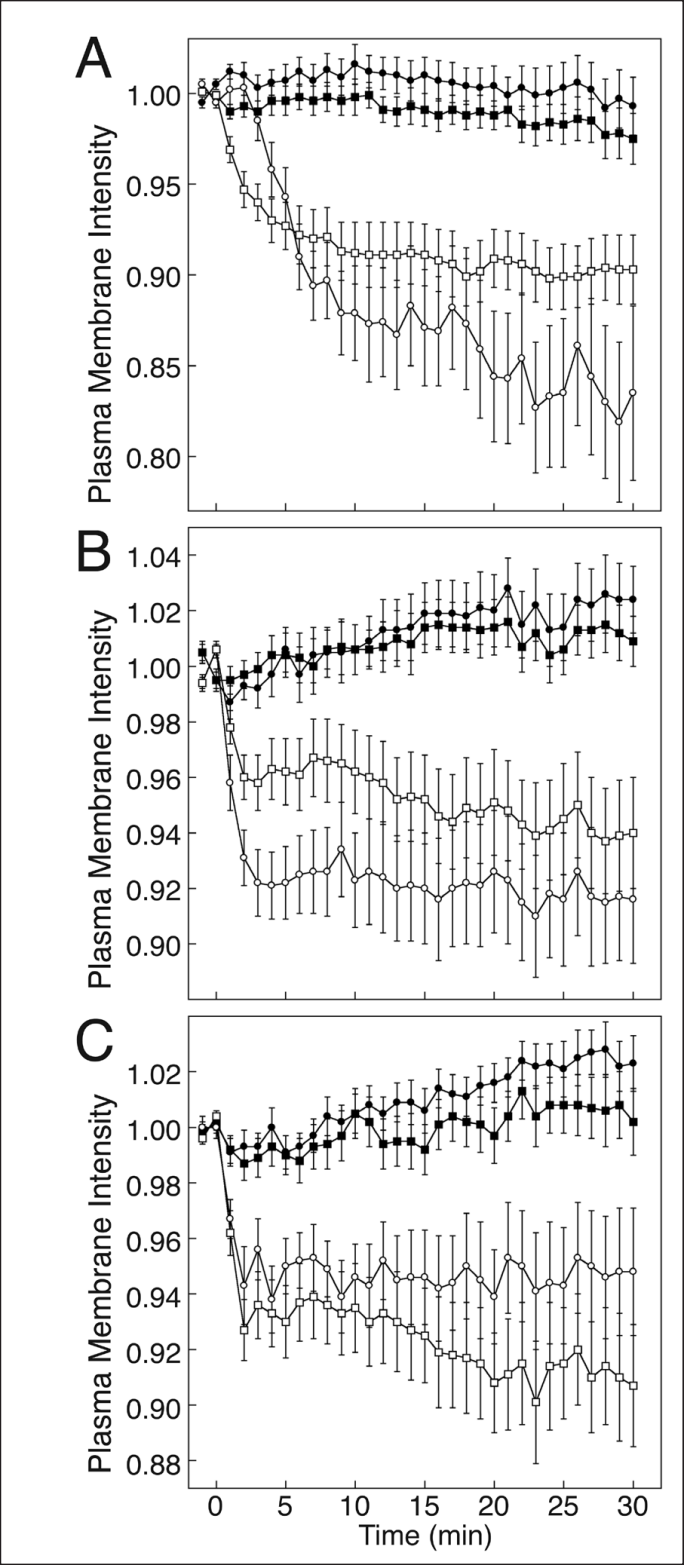
**Quantification of the inhibitory effects of Gα_s_DN3 on internalization of the β_2_AR, Gα_s_, and Gβ_1_γ_7_ from the plasma membrane.** Fluorescent fusion protein internalization responses were measured in HEK-293 cells stimulated with 10 µM isoproterenol following the second time point. Values represent means ± SE. The number of cells analyzed in each case is indicated in parentheses. (A) Gα_s_DN3-CFP blocks isoproterenol-mediated internalization of β_2_AR-GFP. 10^5^ cells were transfected with the following plasmids: Gα_s_DN3-CFP or Gα_s_-CFP, 0.15 µg; Gβ_1_ and Gγ_7_, 0.075 µg each; β_2_AR-GFP, 0.05 µg; mRFP-Mem, 0.0025 µg. Plasma membrane intensity values for β_2_AR-GFP in the presence of Gα_s_-CFP (open circles, 30 cells) and in the presence of Gα_s_DN3-CFP (filled circles, 37 cells), for Gα_s_-CFP (open squares, 30 cells), and for Gα_s_DN3-CFP (filled squares, 37 cells) were determined as described in Methods. (B) Gα_s_DN3-CFP blocks isoproterenol-mediated internalization of Gα_s_-YFP. 10^5^ cells were transfected with the following plasmids: Gα_s_DN3-CFP or Gα_s_-CFP, 0.075 µg; Gα_s_-YFP, 0.075 µg; Gβ_1_ and Gγ_7_, 0.075 µg each; β_2_AR, 0.05 µg; mRFP-Mem, 0.0025 µg. Plasma membrane intensity values are indicated as follows: Gα_s_-YFP in the presence of Gα_s_-CFP (open circles, 29 cells) and in the presence of Gα_s_DN3-CFP (filled circles, 37 cells), Gα_s_-CFP (open squares, 29 cells), and Gα_s_DN3-CFP (filled squares, 37 cells). (C) Gα_s_DN3-CFP blocks isoproterenol-mediated internalization of YFP-N-Gβ_1_/YFP-C-Gγ_7_. 10^5^ cells were transfected with the following plasmids: Gα_s_DN3-CFP or Gα_s_-CFP, 0.15 µg; YFP-N-Gβ_1_ and YFP-C-Gγ_7_, 0.075 µg each; β_2_AR, 0.05 µg; mRFP-Mem, 0.0025 µg. Plasma membrane intensity values are indicated as follows: YFP-N-Gβ_1_/YFP-C-Gγ_7_ in the presence of Gα_s_-CFP (open circles, 25 cells) and in the presence of Gα_s_DN3-CFP (filled circles, 35 cells), Gα_s_-CFP (open squares, 25 cells), and Gα_s_DN3-CFP (filled squares, 35 cells).

**Figure 4:**
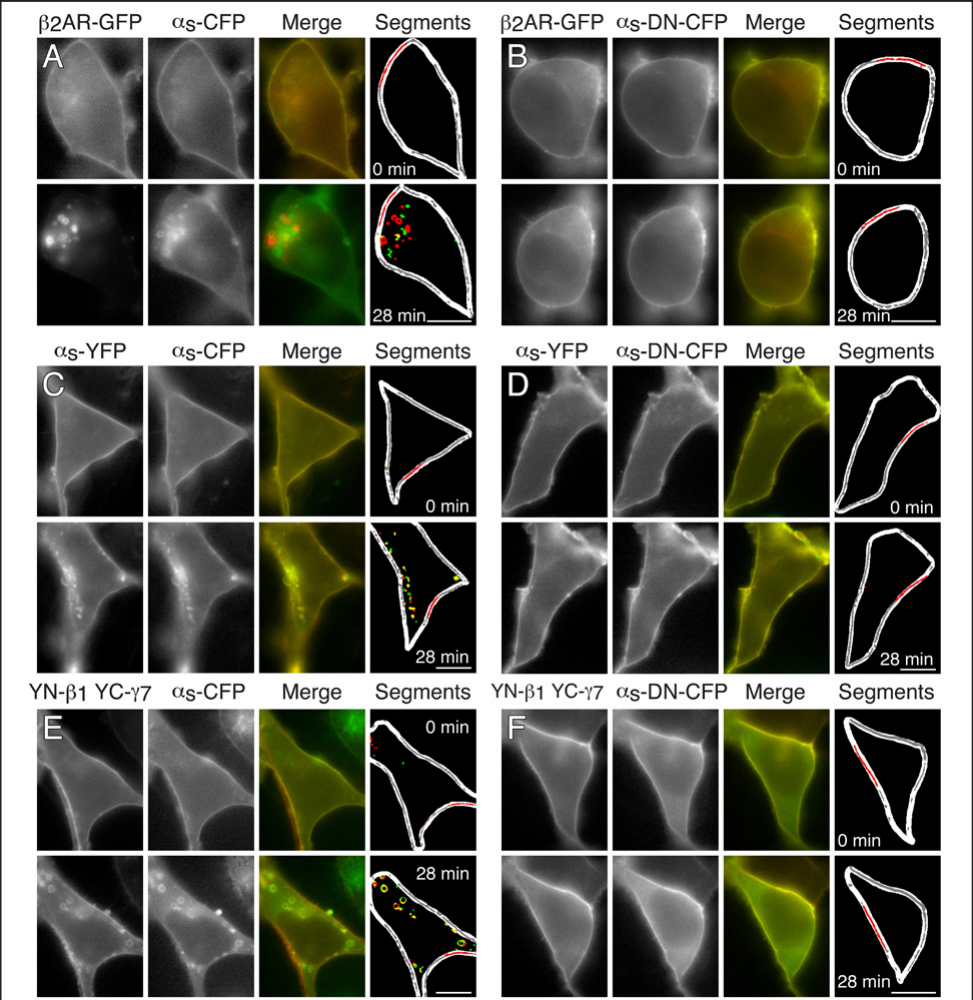
**Representative images showing effects of Gα_s_DN3 on stimulus-dependent internalization of the β_2_AR, Gα_s_, and Gβ_1_γ_7_ in HEK-293 cells.** Top rows of images, before stimulation; bottom rows, 28 minutes after stimulation. (A and B) Gα_s_DN3-CFP blocks isoproterenol-stimulated internalization of β_2_AR-GFP. Cells were transfected with β_2_AR-GFP, Gβ_1_, Gγ_7_, mRFP-Mem, and either Gα_s_-CFP (A) or Gα_s_DN3-CFP (B). To compensate for the increased brightness of the β_2_AR-GFP image after stimulation in (A), restricted ranges of the pixel values of the β_2_AR-GFP images were plotted as follows: 0 min, 0-950; 28 min, 0-3800. (C and D) Gα_s_DN3-CFP does not internalize and blocks isoproterenol-stimulated internalization of Gα_s_-YFP. Cells were transfected with β_2_AR-GFP, Gβ_1_, Gγ_7_, mRFP-Mem, and either Gα_s_-CFP (C) or Gα_s_DN3-CFP (D). (E and F) Gα_s_DN3-CFP blocks isoproterenol-stimulated internalization of YFP-N-Gβ_1_/YFP-C-Gγ_7_. Cells were transfected with β_2_AR, YFP-N-Gβ_1_, YFP-C-Gγ_7_, mRFP-Mem, and either Gα_s_-CFP (E) or Gα_s_DN3-CFP (F). In the merge images and the cytoplasmic regions of the segments images, GFP and YFP fusion proteins are red, Gα_s_-CFP and Gα_s_DN3-CFP are green, and overlap is yellow. In the segments image, the cell border is white, the segmented plasma membrane is gray, the portion of the plasma membrane segment used for analysis of intensity is red, and overlap of vesicle segments is shown in yellow. Vesicles were segmented as described [36]. Plasmid amounts used in the transfections are given in the legend for Fig. 3. α_s_-CFP indicates Gα_s_-CFP, α_s_-DN-CFP indicates Gα_s_DN3-CFP, YN indicates YFP-N, and YC indicates YFP-C. (Bars = 10 µm.)

We tested for effects of Gα_s_DN3 on basal localization and agonist-dependent internalization of the β_2_AR in HEK-293 cells co-expressing β_2_AR-GFP, Gα_s_DN3-CFP, and unlabeled Gβ_1_γ_7_. In cells co-expressing β_2_AR-GFP, Gα_s_-CFP, and unlabeled Gβ_1_γ_7_, both β_2_AR-GFP (Fig. 3A, open circles, Fig. 4A) and Gα_s_-CFP (Fig. 3A, open squares, Fig. 4A) internalized upon stimulation of the cells with the β-adrenergic agonist, isoproterenol. As reported previously [36], the β_2_AR and the G_s_ subunits internalized with different kinetics (Fig. 3A) and did not co-localize during internalization (Fig. 4A). Expression of Gα_s_DN3-CFP did not affect the average intensity of the β_2_AR-GFP signal or the degree to which it associated with the plasma membrane. However, upon stimulation with isoproterenol, neither β_2_AR-GFP (Fig. 3A, filled circles, Fig. 4B) nor Gα_s_DN3-CFP (Fig. 3A, filled squares, Fig. 4B) internalized.

In the presence of Gα_s_DN3-CFP, internalization of both the Gα_s_ and Gβγ subunits of G_s_ was also blocked. In cells co-expressing Gα_s_-YFP, Gα_s_DN3-CFP, and unlabeled β_2_AR and Gβ_1_γ_7_, neither Gα_s_-YFP (Fig. 3B, filled circles, Fig. 4D), nor Gα_s_DN3-CFP (Fig. 3B, filled squares, Fig. 4D) internalized upon stimulation, in contrast to the internalization responses of both Gα_s_-YFP (Fig. 3B, open circles, Fig. 4C) and Gα_s_-CFP (Fig. 3B, open squares, Fig. 4C) that occurred upon stimulation of cells expressing these constructs. Similarly, in cells co-expressing YFP-N-Gβ_1_, YFP-C-Gγ_7_, Gα_s_DN3-CFP, and unlabeled β_2_AR, neither YFP-N-Gβ_1_/YFP-C-Gγ_7_ (Fig. 3C, filled circles, Fig. 4F), nor Gα_s_DN3-CFP (Fig. 3C, filled squares, Fig. 4F) internalized upon stimulation, in contrast to the internalization responses of both YFP-N-Gβ_1_/YFP-C-Gγ_7_ (Fig. 3C, open circles, Fig. 4E) and Gα_s_-CFP (Fig. 3C, open squares, Fig. 4E) that occurred upon stimulation of cells expressing these constructs. These results suggest that, similar to the effect of ZM-241385 on A_2A_R signaling, Gα_s_DN3 blocks G_s_PCR-stimulated Gα_s_ and Gβγ signaling, consistent with the formation of a stable GPCR-G_s_ complex that does not dissociate upon binding of agonist.

### Gα_s_ siRNA and ddA, an adenylyl cyclase inhibitor, decrease TCR-stimulated IL-2 mRNA levels

The results described above demonstrate an inhibitory role of G_s_PCR/G_s_ signaling on TCR-stimulated IL-2 mRNA production, in agreement with numerous previous studies of G_s_PCRs such as the A_2A_R[10,11,12,13], PGE_2_ receptors [14,15], and VIP receptors [16,17]. If the observed potentiating effects of ZM-241385 and Gα_s_DN3 on TCR-stimulated IL-2 mRNA production were simply the result of blocking adenylyl cyclase stimulation by activated Gα_s_, then Gα_s_ siRNA and ddA, an adenylyl cyclase inhibitor, would be expected to have similar potentiating effects.

**Figure 5:**
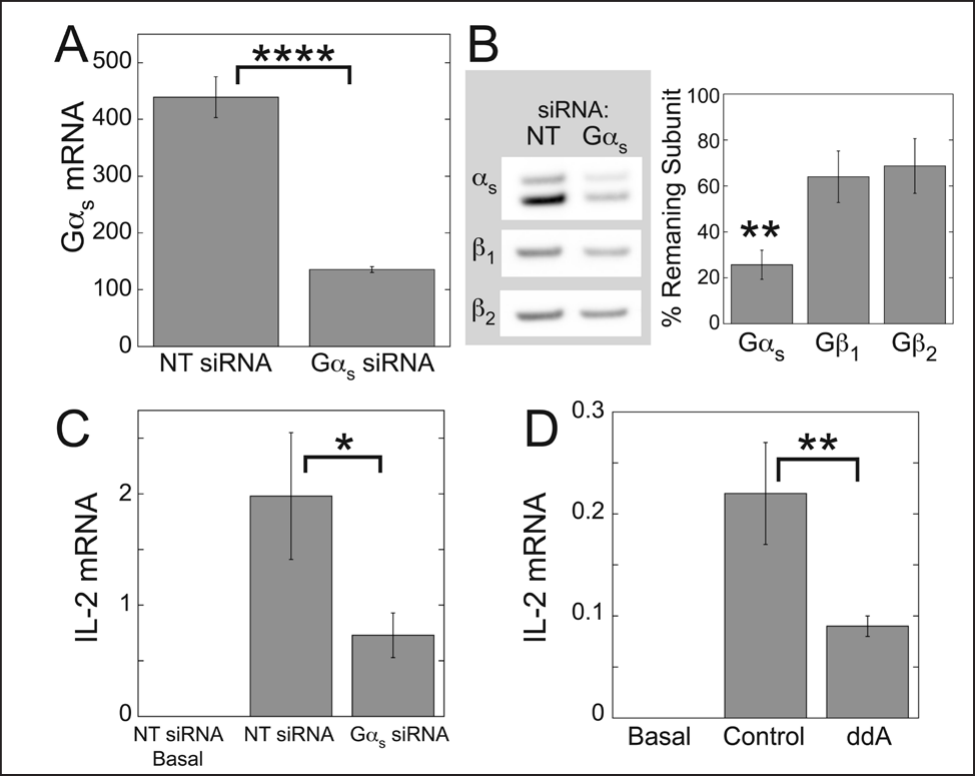
**Gα_s_ siRNA and adenylyl cyclase inhibition with ddA decrease TCR-stimulated IL-2 mRNA levels.** Jurkat cells were nucleofected with Gα_s_ siRNA or NT siRNA as described in Methods (A-C) and stimulated with plate-bound anti-CD3 and soluble anti-CD28 for 3 days (A, C). Gα_s_ siRNA significantly decreased levels of Gα_s_ mRNA (A), Gα_s_ protein (B), and IL-2 mRNA (C). Data for (A) and (C) represent the mean ± SE from 8 experiments. (B) Left, each immunoblot is representative of three immunoblots. Right, quantification of protein expression levels in the presence of Gα_s_ siRNA relative to NT siRNA. Data represent mean ± SE from 3 experiments. (D) Jurkat cells were stimulated with plate-bound anti-CD3 and soluble anti-CD28 for 3 days in the presence or absence of ddA. Data represent the mean ± SE from 17 experiments. mRNA levels were determined by qPCR. ^*^, *p* < 0.05; ^**^, *p* < 0.01; ^****^, *p* < 0.0001.

Expression of Gα_s_ siRNA in Jurkat cells decreased Gα_s_ mRNA to 30% (Fig. 5A) and Gα_s_ protein to 26% (Fig. 5B) of the levels in cells expressing NT siRNA. Gβ_1_ and Gβ_2_ mRNA account for >99% of Gβ mRNA in Jurkat cells [56] and Gα_s_ siRNA caused slight decreases in Gβ_1_ and Gβ_2_ protein expression, but these decreases were not statistically significant (Fig. 5B). Larger and significant decreases in Gβ_1_ and Gβ_2_ protein expression enhanced or had no effect, respectively, on TCR-stimulated IL-2 mRNA levels [56]. Surprisingly, in contrast to the potentiating effects of ZM-241385 and Gα_s_DN3 on TCR-stimulated IL-2 mRNA levels, Gα_s_ siRNA decreased TCR-stimulated IL-2 mRNA to 39% of the value obtained with NT siRNA (Fig. 5C), and ddA decreased TCR-stimulated IL-2 mRNA to 41% of the control value (Fig. 5D).

### Inhibiting adenylyl cyclase decreases TCR-stimulated activity of the IL-2 promoter

**Figure 6:**
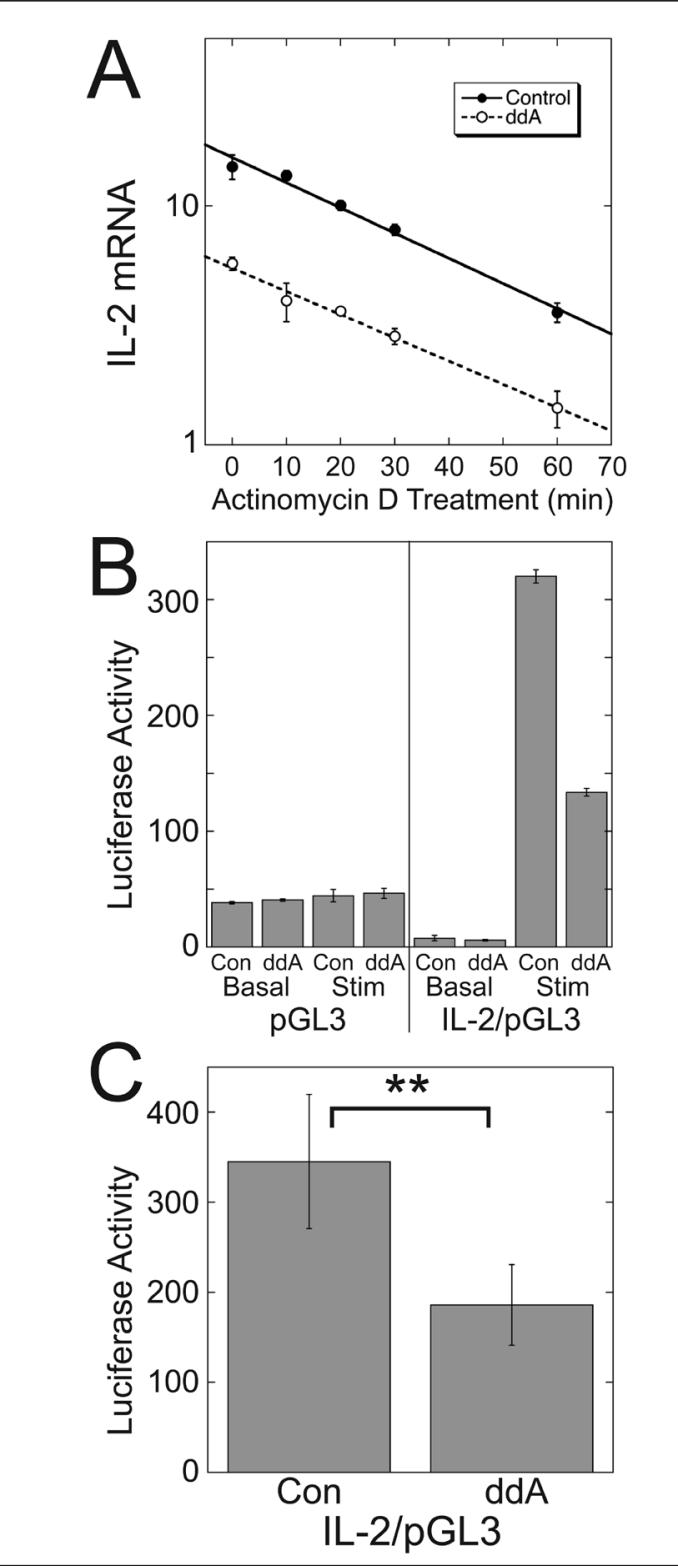
**Inhibiting cAMP production decreases activity of the IL-2 promoter without affecting IL-2 mRNA stability.** (A) ddA does not decrease stability of IL-2 mRNA. After 3 days of TCR stimulation with plate-bound anti-CD3 and soluble anti-CD28 in the presence or absence of ddA, Jurkat cells were incubated for the indicated times with Actinomycin D to inhibit transcription, and the rate of IL-2 mRNA degradation was measured. In both cases, the rates of IL-2 mRNA degradation fit a single exponential. Data represent means ± SD from triplicate determinations from a single experiment representative of 4 experiments. (B) ddA decreases IL-2 promoter activity in a luciferase reporter assay. Jurkat cells were stimulated with plate-bound anti-CD3 and soluble anti-CD28 in the presence or absence of ddA for 3 days following nucleofection with the indicated plasmids. (B) Data represent means ± SD from triplicate determinations from a single assay representative of 6 assays. (C) Data represent the means ± SE of values from stimulated cells expressing IL2/pGL3 from the 6 assays. ^**^, *p* < 0.01.

Inhibiting adenylyl cyclase activity could decrease TCR-stimulated increases in IL-2 mRNA levels by decreasing IL-2 transcription and/or IL-2 mRNA stability. To determine whether inhibition of adenylyl cyclase decreased IL-2 mRNA stability, we measured the half-life of IL-2 mRNA in Jurkat cells stimulated with plate-bound anti-CD3 antibodies and soluble anti-CD28 antibodies for three days and then treated with Actinomycin D to inhibit transcription. ddA did not decrease the stability of IL-2 mRNA (Fig. 6A). The t_1/2_ of IL-2 mRNA from cells treated with ddA (27.30 min, SE = 1.95, N = 4) was the same as that from untreated cells (25.16, SE = 1.87, N = 4).

To test whether inhibiting adenylyl cyclase activity decreased IL-2 transcription, the effect of ddA on IL-2 promoter activity was determined using a luciferase reporter plasmid containing a 1 kB sequence encoding the human IL-2 promoter from -950 to +48 bp. Three days of TCR stimulation increased luciferase activity in the IL-2 reporter plasmid (IL2/pGL3), but not the empty vector (pGL3) (Fig. 6B). ddA reduced the stimulated value of IL2/pGL3 to 55% of the control value (Fig. 6, B and C).

### Gα_s_ siRNA, but not Gα_s_DN3, decreases TCR-stimulated cAMP

The above results demonstrated an important difference between the effects of ZM-241385 and Gα_s_DN3, on the one hand, and Gα_s_ siRNA and ddA, on the other. Namely, the former enhanced TCR-stimulated IL-2 mRNA levels whereas the latter had the opposite effect. Additionally, ZM-241385 and Gα_s_DN3 inhibited signaling of both the Gα_s_ and Gβγ subunits of G_s_, whereas the latter specifically inhibited Gα_s_/cAMP signaling. As both G_s_PCRs and the TCR [32,67,68] can stimulate cAMP increases, these results raised the possibility that the source and context of G_s_ activation can determine whether TCR-stimulated IL-2 production is enhanced or inhibited. As a first step in investigating this possibility, we tested whether TCR-mediated stimulation of cAMP production is mediated by G_s_PCRs.

**Figure 7:**
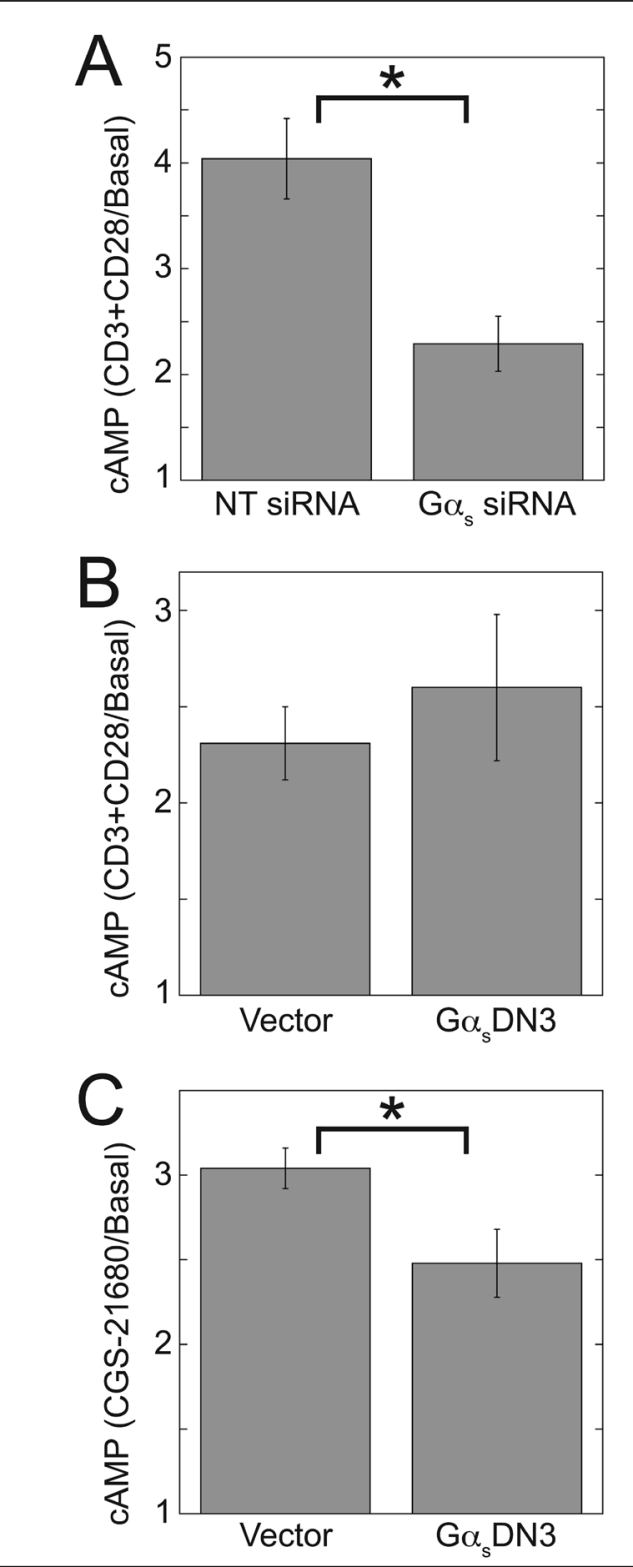
**Gα_s_ siRNA, but not Gα_s_DN3, decreases TCR-stimulated cAMP.** Jurkat cells were nucleofected with the indicated siRNA or plasmids and then assayed for cAMP accumulation as described in Methods. The TCR was stimulated with 2.5 µg/ml plate-bound anti-CD3 and 2.5 µg/ml soluble anti-CD28 (A and B), and the A_2A_R was stimulated with 300 µM CGS-21680 (C). Data in (A) represent the mean ± SE from 3 experiments and data in (B and C) represent the mean ± SE from 9 experiments. ^*^, *p* < 0.05.

Consistent with previous reports of cAMP elevation in response to TCR activation[32,67,68], TCR stimulation increased cAMP accumulation in Jurkat cells (Fig. 7, A and B). Gα_s_ siRNA decreased the TCR-stimulated cAMP increase, indicating that this increase is mediated by activated Gα_s_ (Fig. 7A), in agreement with a previous report showing TCR-stimulated increases of cAMP in lipid rafts, TCR-stimulated recruitment of Gα_s_ to the lipid rafts, and inhibition of TCR-stimulated cAMP increases by inhibitory Gα_s_ antibodies [68]. However, Gα_s_DN3 did not inhibit the TCR-stimulated cAMP increase (Fig. 7B), although it did inhibit A_2A_R-stimulated cAMP increases (Fig. 7C). These results suggest that the TCR stimulates the Gα_s_/cAMP pathway via a mechanism that does not involve a G_s_PCR, which is consistent with a previous study showing that maximal cAMP increases in response to the TCR and to PGE_2_ were additive [67]. These two apparently independent mechanisms of stimulating the Gα_s_/cAMP pathway in T cells could produce differences in the kinetics, amplitude, and/or localization of cAMP increases, which would have implications for the resulting effect on TCR-stimulated IL-2 increases.

### Evidence for an inhibitory effect of cAMP on TCR-stimulated IL-2 mRNA levels after at least 2 days of TCR stimulation

**Figure 8:**
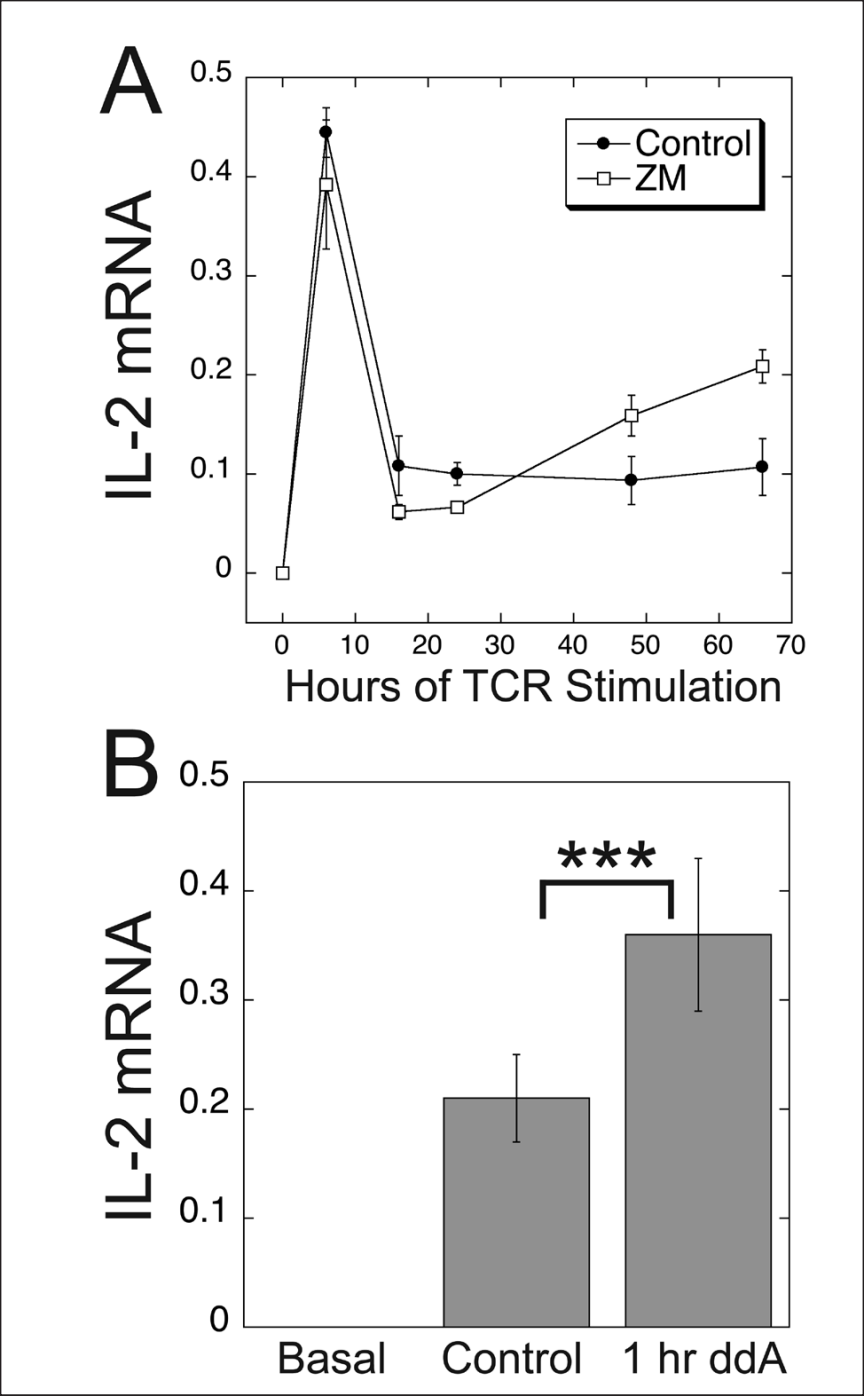
**Evidence for an inhibitory effect of cAMP on TCR-stimulated IL-2 mRNA levels after at least 2 days of TCR stimulation.** (A) The potentiating effect of A_2A_R antagonism was only observed after at least two days of TCR stimulation. IL-2 levels peaked within 24 hours of TCR stimulation and then decreased over the next 48 hours. Jurkat cells were stimulated with plate-bound anti-CD3 and soluble anti-CD28 antibodies in the presence or absence of ZM-241385 (ZM) and IL-2 mRNA levels were determined by qPCR at the indicated times. Data represent the means ± SD from a single experiment that is representative of three such experiments. (B) Stimulation of the TCR for three days followed by one hour of ddA treatment leads to potentiation of TCR-stimulated IL-2 mRNA levels by ddA. After three days of TCR stimulation with plate-bound anti-CD3 and soluble anti-CD28, Jurkat cells were treated with ddA for one hour before determination of IL-2 mRNA levels by qPCR. Data represent the mean ± SE from 14 experiments. ^***^, *p* < 0.001.

As G_s_PCRs such as the A_2A_R function to terminate TCR responses [50], we hypothesized that the duration of TCR-stimulation might influence whether cAMP had an enhancing or inhibiting effect on TCR-stimulated levels of IL-2 mRNA. Ligation of the TCR and CD28 prompts CD4^+^ T cells to secrete IL-2 rapidly, which further enhances their proliferation and survival [69]. However, the levels of IL-2 decrease as the cells start to differentiate [55,70]. Accordingly, we observed an initial peak of IL-2 mRNA within 24 hours of TCR stimulation of Jurkat cells with plate-bound anti-CD3 antibodies and soluble anti-CD28 antibodies that decreased upon further stimulation [56] (Fig. 8A). The enhancing effect of the A_2A_R antagonist, ZM-241385, was only observed after this initial peak, occurring after at least two days of TCR stimulation (Fig. 8A). This result may be explained in part by our observation that during the course of a 3-day stimulation of the TCR, expression of A_2A_R mRNA increased ~4-fold (data not shown), consistent with a previous report that the A_2A_R exhibited increased NFAT-dependent expression upon TCR engagement and that CGS-21680-stimulated cAMP levels were higher in cells that had been stimulated previously with anti-CD3 antibodies [10]. Thus, TCR-stimulated increases in G_s_PCR activity may function as a built-in negative feedback mechanism.

The delayed potentiating effect of ZM-241385 on TCR-stimulated IL-2 mRNA levels might indicate merely that A_2A_R expression was initially limiting. Alternatively, cAMP inhibition might only have an enhancing effect if it occurred after prolonged TCR stimulation. To distinguish between these possibilities, we stimulated the TCR in Jurkat cells for three days and added ddA one hour before harvesting them (Fig. 8B). In contrast to the inhibitory effect of ddA when added from the initiation of TCR stimulation (Fig. 5D), when ddA was added one hour before the cells were harvested and IL-2 mRNA levels were determined, TCR-stimulated IL-2 mRNA levels were enhanced, consistent with an inhibitory effect of cAMP at this stage of TCR-stimulation (Fig. 8B). These results suggest that TCR-stimulated changes in the T cell (see Discussion) influence whether cAMP plays an enhancing or inhibitory role in regulation of IL-2 mRNA levels.

## Discussion

**Figure 9:**
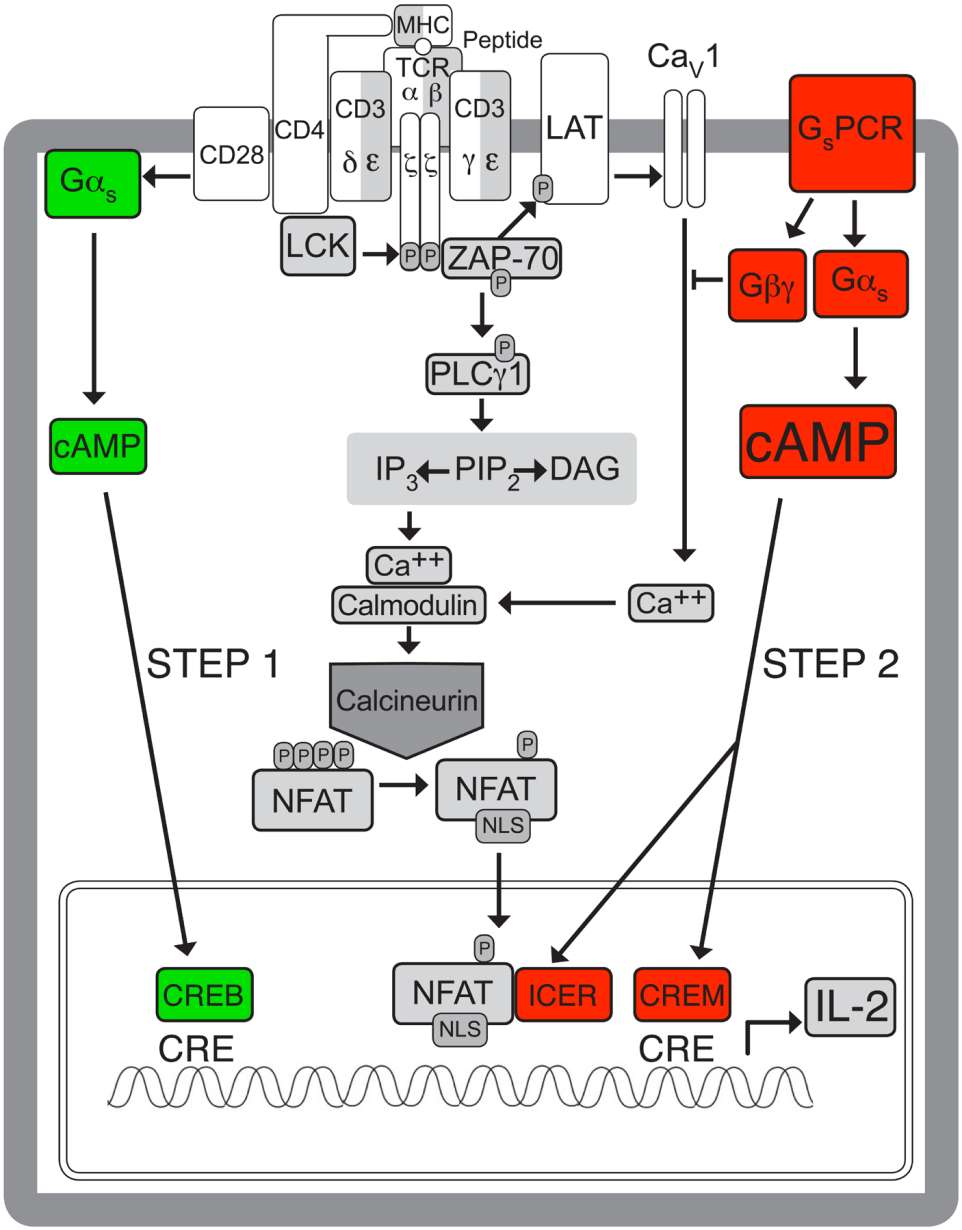
**Model of how the source and context of activated Gα_s_ and cAMP may determine whether they enhance or inhibit TCR-stimulated IL-2 transcription.** Interactions between the TCR and peptide-major histocompatibility complex (MHC) lead to recruitment of CD4 and its associated kinase, p56-Lck, which phosphorylates tyrosine residues in the cytoplasmic tails of the TCR subunits, leading to recruitment and phosphorylation of the tyrosine kinase, ZAP-70. CD28 co-stimulation provides an additional signal that is needed for complete T cell activation and regulation of IL-2 production [46]. ZAP-70 and p56-Lck then phosphorylate and activate numerous downstream target proteins, including PLC-γ, leading to Ca^2+^ increases and activation of a variety of downstream pathways including translocation of NFAT to the nucleus and activation of IL-2 transcription [77] (black and white pathway). Gα_s_ stimulated by a mechanism that does not involve G_s_PCRs, but which could potentially involve the TCR, enhances TCR-stimulated IL-2 transcription by a mechanism that may involve binding of pCREB to the CRE site of the IL-2 promoter [68,78,79,80] during the initial stages of TCR stimulation (green pathway, Stimulatory Step 1). In contrast G_s_PCRs decrease TCR-stimulated IL-2 transcription, potentially by utilizing both Gα_s_ and Gβγ signaling in cells that have been exposed to at least two days of TCR stimulation (red pathway, Inhibitory Step 2). The inhibitory G_s_PCR/Gα_s_/cAMP pathway may involve binding of CREM, which gradually replaces pCREB, to the CRE site of the IL-2 promoter [79] or the formation of NFAT/ICER complexes on NFAT/AP-1 composite sites in the IL-2 promoter [81], leading to repression of transcription (Inhibitory Step 2). Previous studies suggest that cAMP increases stimulated by the TCR are smaller and more transient than those stimulated by G_s_PCRs, as depicted by the relative sizes of the cAMP symbols, and this may contribute to the opposite effects on IL-2 transcription. Simultaneously, Gβγ may inhibit TCR-stimulated IL-2 transcription [56] by decreasing TCR-stimulated Ca^2+^ increases through Ca_v_1 channels (Inhibitory Step 2), which are activated by the TCR by an unknown mechanism [72]. Ca^2+^-calmodulin-activated calcineurin dephosphorylates NFAT, exposing a nuclear localization sequence (NLS) and leading to nuclear translocation.

The effect of cAMP on IL-2 production in T cells has generally been thought to be inhibitory[10,11,12,13,14,15,16,17,22,24], although there is also some evidence to the contrary [32,33]. The results presented here demonstrate that the effect of inhibiting cAMP increases on IL-2 mRNA levels in TCR-stimulated CD4^+^ T cells depends on the means by which this is accomplished. These results support both an inhibitory role for G_s_PCRs and a stimulatory one for Gα_s_ and cAMP in the regulation of TCR-stimulated IL-2 mRNA levels (Fig. 9). The source of the activated Gα_s_ that plays a positive role has not been identified yet, but the TCR is one possibility, as it appears to stimulate cAMP synthesis via a non-canonical mechanism that involves activation of Gα_s_, but not G_s_PCRs, and as discussed below, the cAMP increases stimulated by the TCR compared to G_s_PCRs are likely to be more modest and transient, characteristics associated with an enhancing effect on TCR-stimulated IL-2 [32,33].

Based on prior results [16,17,22] and those presented here, the context of Gα_s_/cAMP signaling appears to be an important determinant of its effect on IL-2 production by activated T cells. The presence or absence of uninhibited Gβγ signaling is one important contextual difference between TCR-stimulated T cells in which G_s_PCRs versus Gα_s_ or adenylyl cyclase are blocked. Whereas knocking down Gα_s_ expression and inhibiting adenylyl cyclase activity each decreased levels of TCR-stimulated IL-2 mRNA, potentiation of these mRNA levels was obtained when both Gα_s_ and Gβγ signaling were blocked, which is important in light of our previous observation that inhibition of Gβγ alone with the small molecule inhibitor, gallein, enhanced TCR-stimulated IL-2 transcription [56]. This could indicate that inhibition of both the Gα_s_ and Gβγ components of G_s_ is necessary to obtain a stimulatory effect, and that simultaneous Gβγ signaling determines the effect of Gα_s_/cAMP signaling on TCR-stimulated IL-2 mRNA levels in a manner analogous to B-Raf, which is a cell type-specific molecular switch that determines whether cAMP has a stimulatory or inhibitory effect on MAPK activity in central nervous system parenchymal cells [4]. Alternatively, inhibiting the Gβγ component of G_s_ alone may be sufficient to potentiate TCR-stimulated IL-2 transcription, even without a decrease in Gα_s_ activity and cAMP levels. As gallein blocks the interactions of Gβγ with downstream effectors rather than exclusively inhibiting Gβγ derived from a particular G protein heterotrimer such as G_s_ [_59_,_71_], it is currently not possible to distinguish between these two possibilities.

One potential mechanism by which simultaneous Gβγ activation could influence the effect of Gα_s_/cAMP signaling on TCR-stimulated IL-2 transcription is via inhibition of TCR-stimulated increases in intracellular Ca^2+^ levels (Fig. 9). We determined previously that inhibiting Gβγ led to increased levels of intracellular Ca^2+^ in TCR-stimulated CD4^+^ T cells [56]. The mechanism for this effect of Gβγ inhibition remains to be determined, but may involve L-type voltage-dependent Ca^2+^ (Ca_V_1) channels, which are expressed in primary human T cells and Jurkat cells, are activated by the TCR by an unknown mechanism, rather than by T cell depolarization [72], and are important for Ca^2+^-mediated NFAT translocation to the nucleus and IL-2 production [72,73] (Fig. 9). Gβγ can block activation of Ca_V_1 channels [74,75,76] and gallein can prevent this effect of Gβγ [76]. TCR-stimulated signaling involves increases in intracellular Ca^2+^ in response to IP_3_ generated by activated PLC-γ, resulting in activation of a variety of downstream pathways including translocation of NFAT to the nucleus and activation of IL-2 transcription [77] (Fig. 9). Whereas G_s_PCR stimulated G_s_ might simultaneously increase cAMP via Gα_s_ and decrease Ca^2+^ via Gβγ (Fig. 9), activation of Gα_s_/cAMP signaling alone might potentiate TCR-stimulated Ca^2+^ increases, as has been demonstrated for transient adhesion-dependent cAMP increases [31].

Most of our experiments involved antagonizing or blocking G_s_PCR signaling, knocking down Gα_s_ expression, or inhibiting adenylyl cyclase activity from the initial onset of a 3-day interval of TCR stimulation. The observed negative effects of blocking Gα_s_/cAMP signaling are consistent with the decreased T cell functioning seen in knockout animals for Gα_s_ [26] and for the AC7 isoform of adenylyl cyclase [27], in which cAMP signaling is blocked before the initiation of TCR stimulation. In contrast, our data showing that potentiation of IL-2 mRNA levels by ZM-241385 required at least two days of TCR stimulation and that addition of ddA after three days of TCR stimulation enhanced IL-2 mRNA levels (Fig. 8) suggest that the inhibitory effects of cAMP on IL-2 transcription occur only after the initiation of TCR stimulation. Of note, the potentiating effect of Gβγ inhibition on IL-2 transcription required continuous Gβγ inhibition for at least two days of TCR stimulation [56], implicating a delayed effect of both the Gα_s_ and Gβγ components of G_s_PCRs relative to initiation of TCR stimulation. Previous reports suggest a possible mechanism for differential effects of cAMP on TCR-stimulated IL-2 depending on the duration of TCR stimulation. TCR-stimulation initially leads to phosphorylation of CRE-binding protein (CREB), which then recruits p300 and CREB-binding protein (CBP) and binds to the IL-2 promoter to activate transcription [68,78,79,80] (Activating Step 1 in Fig. 9). Later it is replaced by cAMP response element (CRE) modulator (CREM), which exerts an inhibitory effect on IL-2 transcription that occurs after the initial increase in TCR-stimulated IL-2 levels [79] (Inhibitory Step 2 in Fig. 9). cAMP also inhibits IL-2 transcription via inducible cAMP early represser (ICER), a cAMP inducible CREM family member that can form NFAT/ICER complexes on several NFAT/AP-1 composite sites in the IL-2 promoter leading to repression of transcription [81] (Inhibitory Step 2 in Fig. 9). ICER mRNA [82] and protein [81] were not detected until after three hours of exposure of human medullary thymocytes to forskolin treatment. Furthermore, a study of the kinetics of inhibition of IL-2 transcription by forskolin demonstrated a delay in inhibition of IL-2 mRNA accumulation that correlated with a delay in inhibition of NF-κB activity [83]. Therefore, our results, taken together with these previous reports, are consistent with a stimulatory role for cAMP during the early stages of T cell activation that would be blocked by Gα_s_ siRNA and ddA.

Based on the ability of TCR stimulation to elevate cAMP by a mechanism that is inhibited by Gα_s_ siRNA, but not by Gα_s_DN3, Gα_s_ activation by the TCR appears to involve a non-canonical mechanism that does not involve a G_s_PCR. There is precedent for non-GPCR-dependent G protein activation and such a mechanism may operate in T cells [84]. For instance, the TCR signals to integrins [85], integrins can activate Gα_s_, leading to translocation of phosphorylated CREB to the nucleus [86], and transient adhesion-dependent cAMP increases are stimulatory to TCR signaling [31]. Additionally, Ric-8B [87] and Cysteine String Protein (CSP) [88] can catalyze nucleotide exchange on free Gα_s_-GDP.

Our results showing that TCR-stimulated cAMP increases do not appear to involve G_s_PCRs, which inhibit TCR-stimulated IL-2 production, raise the possibility that TCR-stimulated cAMP plays a positive role in IL-2 transcription. One relevant characteristic that distinguishes the cAMP responses stimulated by the TCR versus G_s_PCRs is the differences in amplitude of the cAMP increases. Reported increases in cAMP in response to TCR stimulation were ~2-fold [89] or 4–6-fold [67] rather than the ~13-fold increase induced by PGE_2_ [90]. The levels of cAMP that we measured after 40 minutes of stimulation of either the TCR (Fig. 7, A and B) or the A_2A_R (Fig. 7C) were similar. However, increases in the expression of A_2A_R mRNA in response to TCR stimulation (unpublished) [10] suggest that levels of A_2A_R-stimulated cAMP are likely to be greater after several days of TCR stimulation. In contrast, expression of the TCR on the cell surface, as determined by flow cytometry, was ~4-fold lower after a 3-day TCR stimulation than in unstimulated cells (data not shown), similar to a previous report in which co-stimulation of naïve T cells with antigen and anti-CD28 for 10 hours resulted in down-regulation of ~90% of the TCRs [47]. Taken together, these results suggest that by three days of TCR stimulation cAMP increases in response to A_2A_R stimulation would be greater than those due to TCR stimulation.

Another variable that might determine the directionality of the effect of Gα_s_/cAMP signaling on TCR-stimulated IL-2 mRNA levels is the kinetics of the cAMP response. Recently it has become possible to monitor cAMP increases in real time in single cells using a FRET-based cAMP sensor, AKAR2, which detects conformational changes induced by PKA phosphorylation [91]. Use of this probe in T cells showed that adhesion-dependent cAMP increases peaked in less than 2 minutes and returned to baseline within 10 minutes [31]. Whereas these transient cAMP increases were stimulatory to TCR signaling, sustained increases in response to forskolin were inhibitory [31]. Anti-CD3-stimulated cAMP increases with similar transient kinetics have been reported in T cell populations. Stimulation of Jurkat cells with anti-CD3 produced a cAMP increase that peaked at 1 minute [67], and antibody-mediated cross-linking of anti-CD3 antibodies on primary human T lymphocytes produced peak cAMP levels within 2 minutes [89]. In contrast, the kinetics of G_s_PCR-stimulated cAMP increases appears to be somewhat slower. For instance, PGE_2_-stimulated cAMP increases peaked at 5 minutes [90]. Taken together with previous reports demonstrating a positive correlation between small and transient cAMP increases and IL-2 production [32,33], these observations are consistent with a positive effect on TCR-stimulated IL-2 production of modest and transient cAMP increases in response to a non-canonical Gα_s_ activator such as the TCR itself in contrast to the negative effect of G_s_PCR stimulation (Fig. 9).

## Conclusions

Inhibition of G_s_PCR signaling in TCR-stimulated CD4^+^ T helper cells enhanced TCR-stimulated increases in IL-2 mRNA, but knocking down Gα_s_ expression, or inhibiting adenylyl cyclase activity had the opposite effect. As inhibiting G_s_PCRs blocks both the Gα_s_ and Gβγ components of G_s_, and inhibiting Gβγ alone enhances TCR-stimulated increases in IL-2 mRNA, the presence of simultaneously activated Gβγ may determine the effect of activating the Gα_s_/cAMP pathway. Additionally, the TCR appears to stimulate cAMP synthesis via a non-canonical mechanism that involves activation of Gα_s_, but not G_s_PCRs. As prior reports showed that TCR-stimulated cAMP increases are smaller and more transient than those induced by G_s_PCRs, and modest and transient cAMP increases have been associated with enhancement of T cell function, the TCR may be a source of Gα_s_/cAMP signaling that plays a positive role in IL-2 transcription. Finally, as potentiation of IL-2 mRNA levels by upon A_2A_R antagonism required at least two days of TCR stimulation, and inhibition of adenylyl cyclase after three days of TCR stimulation enhanced IL-2 mRNA levels, the stage of T cell activation and differentiation appears to determine the effect of Gα_s_/cAMP signaling on TCR-stimulated IL-2 mRNA levels.

## Competing Interests

The authors declare that they have no competing interests.
